# Association between prescription drugs and all‐cause mortality risk in the UK population

**DOI:** 10.1111/acel.14334

**Published:** 2024-10-04

**Authors:** Jonas Morin, Yves Rolland, Heike A. Bischoff‐Ferrari, Alejandro Ocampo, Kevin Perez

**Affiliations:** ^1^ EPITERNA Epalinges Switzerland; ^2^ IHU HealthAge, UMR CERPOP 1295, CHU Toulouse Toulouse France; ^3^ Department of Geriatric Medicine and Aging Research University of Zurich Zurich Switzerland; ^4^ Department of Biomedical Sciences, Faculty of Biology and Medicine University of Lausanne Lausanne Switzerland

**Keywords:** aging, all‐cause mortality, biobank, lifespan, medications, mortality risk, prescription drugs, repurposing

## Abstract

Although most drugs currently approved are meant to treat specific diseases or symptoms, it has been hypothesized that some might bear a beneficial effect on lifespan in healthy older individuals, outside of their specific disease indication. Such drugs include, among others, metformin, SGLT2 inhibitors and rapamycin. Since 2006, the UK biobank has recorded prescription medication and mortality data for over 500′000 participants, aged between 40 and 70 years old. In this work, we examined the impact of the top 406 prescribed medications on overall mortality rates within the general population of the UK. As expected, most drugs were linked to a shorter lifespan, likely due to the life‐limiting nature of the diseases they are prescribed to treat. Importantly, a few drugs were associated with increased lifespans, including notably Sildenafil, Atorvastatin, Naproxen and Estradiol. These retrospective results warrant further investigation in randomized controlled trials.

## Graphical abstract

The UK Biobank has collected data on prescription medications and mortality for over 500,000 participants aged 40–70 years. We analyzed the effects of the top 406 prescribed medications on overall mortality rates in the general population. Most drugs were linked to a shortened lifespan, likely due to the life‐limiting nature of the diseases they are prescribed to treat. However, a few medications, notably Sildenafil, Atorvastatin, Naproxen, and Estradiol, were associated with increased lifespans.

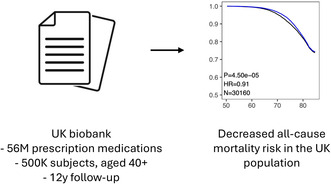

**Abbreviations**
ACE: angiotensin‐converting enzyme
CI: confidence interval
COPD: chronic obstructive pulmonary disease
CoxPh: Cox proportional hazard
CVD: cardiovascular disease
DVT: deep vein thrombosis
ERT: estrogen replacement therapy
FDR: false‐discovery rate
HR: hazard ratio
ICD: International Statistical Classification of Diseases
MET: metabolic equivalent task
NNCM: Nearest Neighbor Covariate Matching
PDE5: phosphodiesterase type 5 inhibitor
RCT: randomized controlled trials
SD: standard deviation
SGLT2: sodium/glucose cotransporter 2
UKBB: UK biobank

## 1 INTRODUCTION

With an increasingly aging population, aging poses a significant challenge on society and health care systems (Cristea et al., 2020), aging being the major risk factor for all age‐related diseases. With aging also comes a slew of decline in functional ability (mobility, cognition, and immunity), and a dramatic increase in mortality risk. Promoting healthy aging through primary prevention and lifestyle modifications (avoidance of smoking and alcohol, exercise, diet, sleep) is a well‐demonstrated but also complicated strategy to implement. It is then also important to identify drugs that can prevent the functional decline associated with aging, delay the occurrence of age‐related diseases, and reduce mortality risk in the elderly. Medications are usually associated with a higher mortality rate. This relation can be explained, in part, by the potential side‐effects of the drugs, but mainly because the underlying diseases the drugs are prescribed to treat have themselves an increased mortality risk, that the drug is not able to counteract. Furthermore, increased medication in the population is generally seen as an indicator of poor health, and polypharmacy negatively impacts life expectancy in old people (Chang et al., 2020; Davies et al., 2022).

However, following the “Geroscience hypothesis” (Sierra, 2016), some long‐established drugs are being proposed to be tested for their protective effect on aging and mortality in a healthy aging population, including metformin, rapamycin, sodium/glucose cotransporter 2 (SGLT2) inhibitors, acarbose, angiotensin‐converting enzyme (ACE) inhibitors, or senolytics (Kulkarni et al., 2022; Rolland et al., 2023). Aspirin was notably tested for its beneficial effect on survival in a large, randomized trial in older adults, but with negative results (McNeil et al., 2018). In a retrospective study, metformin was shown to provide increased overall survival, even compared to non‐diabetic control subjects (Bannister et al., 2014). This finding suggests that other approved drugs could be repurposed to decrease mortality risks in healthy middle‐aged or elderly adults. Yet, one of the main challenges is that drugs are typically prescribed to patients for a specific disease indication, who may already have an increased mortality risk due to this underlying disease, thus confounding the potential beneficial effect on lifespan.

Previous efforts have been made to evaluate the effects of medications on mortality in the general population, but most studies tend to focus on specific classes of medications used for a particular disease (Araldi et al., 2023; Hasan & Hasan, 2017; Nowak et al., 2022; Tiihonen et al., 2016). Notably, the effect of specific medications on mortality risk has been assessed previously for cancer treatments in cancer patients (Miller et al., 2022), statins in patients with or at risk for cardiovascular diseases (Andersson et al., 2023; Collaboration, CTT), 2010; Nowak et al., 2022), anti‐psychotic and anti‐depressant drugs in schizophrenic patients (Li et al., 2024; Tiihonen et al., 2016), diabetic drugs in diabetic patients (Araldi et al., 2023; Bannister et al., 2014; Mohammed et al., 2021; Stevenson‐Hoare et al., 2023). To date, to the best of our knowledge, a study that comprehensively looks at a wide range of commonly prescribed medications and their association with mortality in the general population is missing.

The UK Biobank (UKBB) is a large‐scale biomedical database and research resource containing de‐identified genetic, lifestyle and health information from half a million UK participants. More precisely, it contains prescription medication data, together with mortality data in the general UK population, in adults aged 37–73, for over 500′000 subjects. We used this resource to assess the effect of medication on mortality risk in the general population for 406 of the most prescribed drugs in the UK. Moreover, we also analyze dose–response effects, gender specific effects, and medication class effects.

## 2 RESULTS

We obtained data from the UKBB on October 24, 2023, including prescription medication records, mortality data, comorbidities, and lifestyle factors. The UKBB study included 501′169 participants recruited between 37 and 73 years old. Data on 56′213′338 prescriptions were available for 222′058 participants. We excluded participants for whom clinical data was unavailable, as well as accidental or self‐inflicted deaths. To identify potential confounders, we first assessed what factors were contributing the most to survival in the general population. 8.4% of patients died, during a median follow‐up of 13.8 years. The age at recruitment was 57yo. (SD 8yo.). 46% were men, 8% had a cancer diagnosis, 5% had diabetes, 10% were current smokers (Table S1). Not surprisingly, among the factors that were available for analysis (Methods), current smoking (HR 2.00, CI 1.94–2.06), cancer diagnosis (HR 1.88, CI 1.83–1.94), diabetes (HR 1.65, CI 1.60–1.70), male gender (HR 1.64, CI 1.60–1.67), and older age at recruitment (HR 1.72, CI 1.69–1.76) were associated with reduced lifespan (Figure S1; Data S1).

After harmonization (Methods, Data S5), we found 406 distinct drugs prescribed to more than 500 patients, for a duration of at least 3 months (from the date between first and last use). The three most prescribed drugs in the dataset were amoxicillin (*N* = 73,371), simvastatin (*N* = 45,776), and omeprazole (*N* = 44,100) (Data S2). For each drug we then assessed one by one the survival of patients taking the drug, compared to health‐matched controls not taking the drug. The matching was based on covariates identified above to have the strongest impact on survival, namely current smoking, cancer diagnosis, diabetes, gender, age at recruitment (Methods). The cohort's health characteristics varied greatly depending on the different prescription drugs. For example, Atorvastatin patients had a higher age at recruitment (61yo. vs. 57yo. for the whole cohort), higher percentage of males (58% vs. 46%), and high percentage of diabetes (15% vs. 5%). The patient matching strategy was therefore critical to be able to correctly assess the effect of each drug (Figure S2).

**FIGURE 1:**
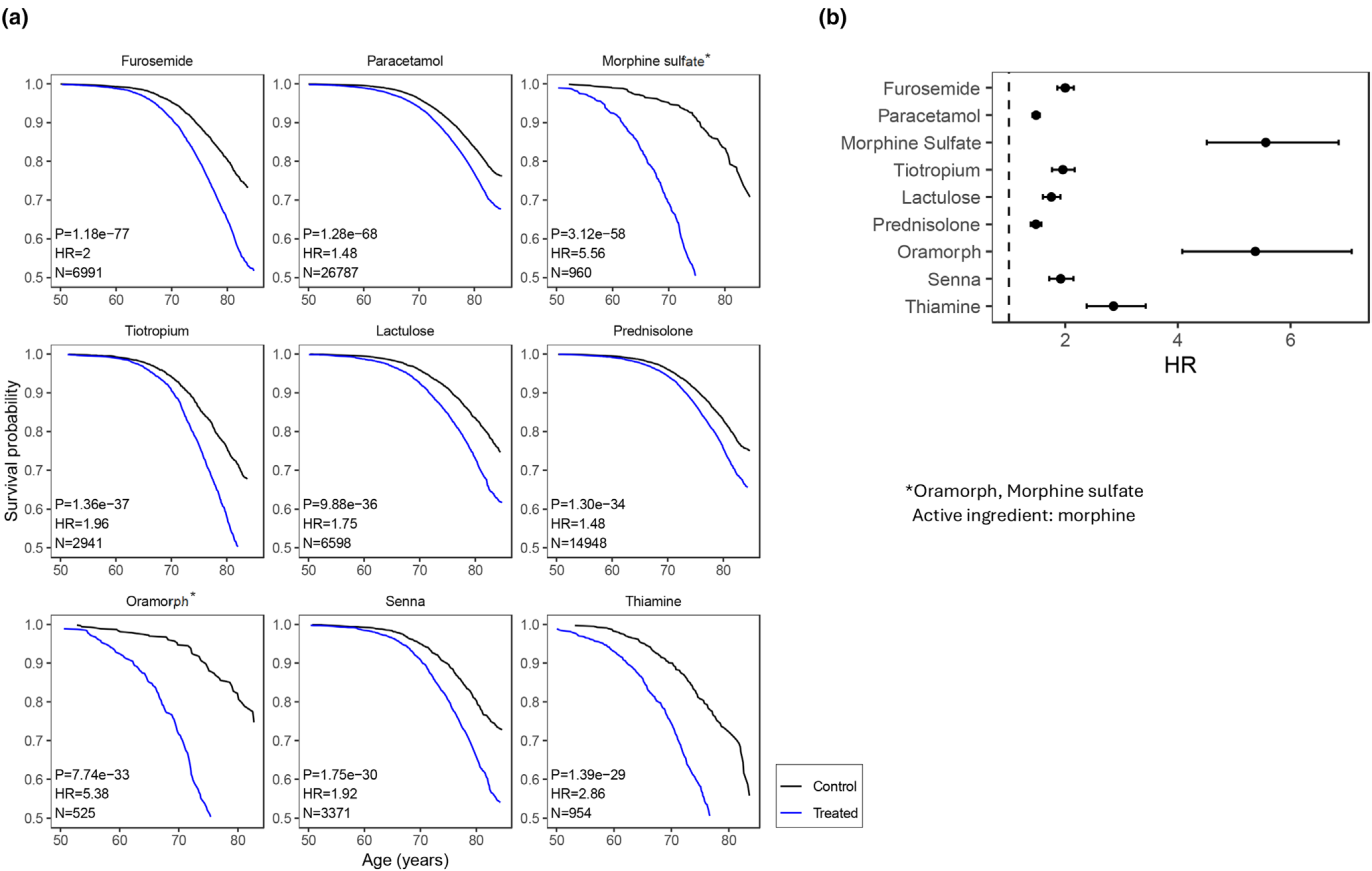
Prescription drugs associated with increased mortality. Top 9 prescription drugs negatively impacting lifespan, ranked by *p*‐value in the Cox Proportional Hazards (CoxPH) model. (a) Survival curves. Survival probability vs. Age (years). N: Sample size (treated), Hazard‐Ratio (HR), >1 for worse survival, *p*‐value of CoxPH model. Treated group in blue, control in black (b) HR with confidence interval 95%. Dotted line HR = 1.

**FIGURE 2:**
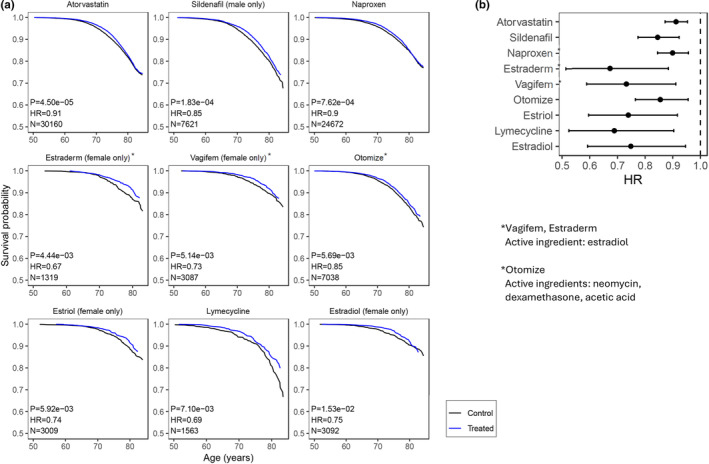
Prescription drugs associated with decreased mortality. Top 9 prescription drugs positively impacting lifespan, ranked by *p*‐value in the Cox Proportional Hazards (CoxPH) model. (a) Survival curves. Survival probability vs. Age (years). N: Sample size (treated), Hazard‐Ratio (HR), >1 for worse survival, *p*‐value of CoxPH model. Treated group in blue, control in black (b) HR with confidence interval 95%. Dotted line HR = 1.

Out of the 406 drugs studied, 169 had a significant effect on lifespan, after multiple comparison correction (FDR <0.05). From these 169, as expected, the majority (*N* = 155, 92%) were associated with increased mortality (Figure 1; Data S2; Table S2). These included notably opioids like Morphine sulfate (HR 5.56, CI 4.51–6.86) and Oramorph (HR 5.38, CI 4.08–7.09), the diuretic Furosemide (HR 2.00, CI 1.86–2.15), pain medication Paracetamol (HR 1.48, CI 1.42–1.55) or chronic obstructive pulmonary disease treatment Tiotropium (HR 1.96, CI 1.77–2.17). Importantly, we identified 14 drugs that were associated with increased lifespan, compared to health matched controls (Figure 2; Data S2 and Table S2), independently of current smoking, cancer diagnosis, diabetes, gender, and age at recruitment. These included notably the statin Atorvastatin (HR 0.91, CI 0.87–0.95), the PDE5 inhibitor Sildenafil (HR 0.85, CI 0.78–0.93), the anti‐inflammatory drug Naproxen (HR 0.90, CI 0.85–0.96), and the estrogen related drugs Estraderm (HR 0.67, CI 0.51–0.88), Vagifem (HR 0.73, CI 0.59–0.91), Estriol (HR 0.74, CI 0.60–0.92) and Estradiol (HR 0.75, CI 0.59–0.95). Others included, Lymecycline, Otomize, Marvelon (desogestrel, ethinylestradiol), and the vaccines Avaxim, and Revaxis.

**FIGURE 3:**
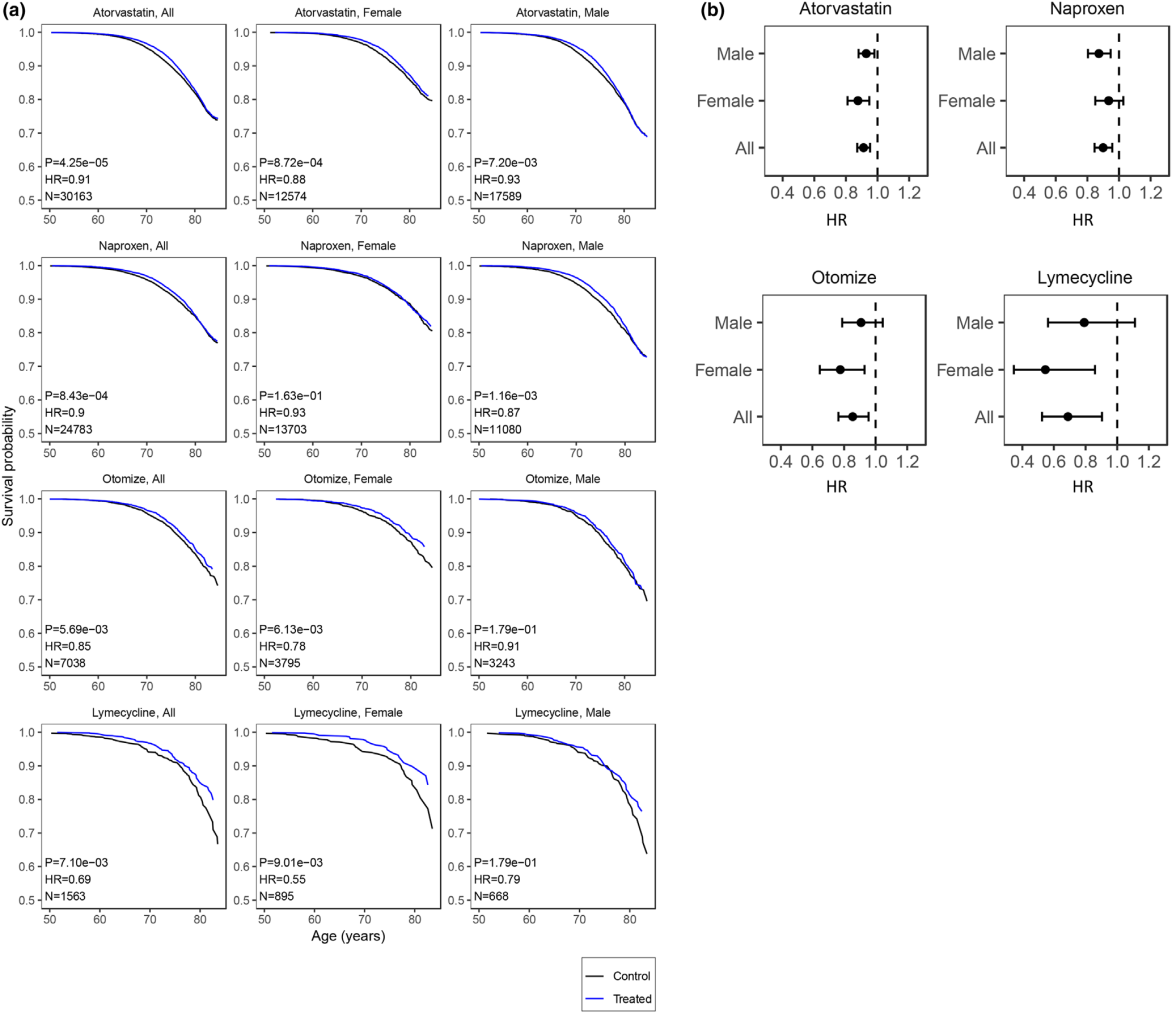
Gender specific effects of prescription drugs associated with decreased mortality. Effect in both (All), female or male only of selected drugs on survival. (a) Survival curves. Survival probability vs. Age (years). N: Sample size (treated), Hazard‐Ratio (HR), >1 for worse survival, *p*‐value of CoxPH model. Treated group in blue, control in black (b) HR with confidence interval 95%. Dotted line HR = 1.

Estrogen related drugs are approved for women only, while PDE5i are for men only, therefore it was possible to look at their effect in one gender only. For drugs approved for both genders, we sought to investigate whether the effect on lifespan was gender‐specific or consistent between men and women. Interestingly Atorvastatin increased lifespan, in both males (HR 0.93, CI 0.88–0.98) and females (HR 0.88, CI 0.81–0.95). Conversely for Naproxen the protective effect was more pronounced in men (HR 0.87, CI 0.80–0.95) than in women, and for Otomize more pronounced in women (HR 0.78, CI 0.65–0.93) than in men (Figure 3; Data S2).

**FIGURE 4:**
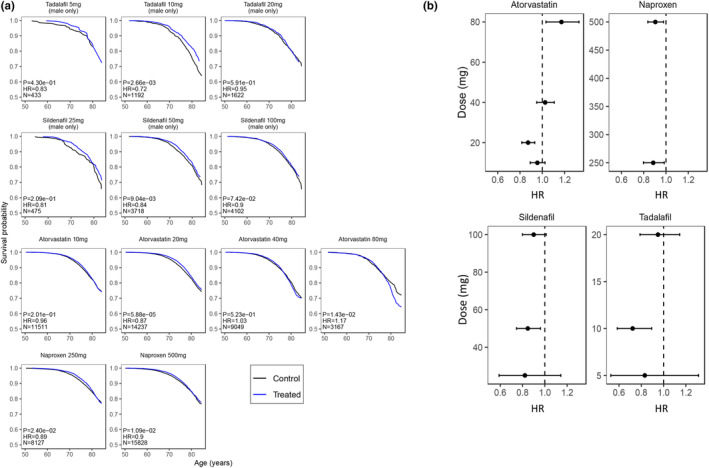
Dose–response effects of prescription drugs associated with decreased mortality. Effect at multiple concentrations (mg) of selected drugs on survival. (a) Survival curves. Survival probability vs. Age (years). N: Sample size (treated), Hazard‐Ratio (HR), >1 for worse survival, *p*‐value of CoxPH model. Treated group in blue, control in black (b) HR with confidence interval 95% in X‐axis. Dose (mg) in Y‐axis. Dotted line HR = 1.

Additionally, we tested the effect of mortality of prescribed drugs, depending on the dosage (Figure 4; Data S3). For Atorvastatin, we observed a *J*‐shaped dose–response effect, with no effect on mortality at 10 mg (HR 0.96, CI 0.89–1.02), a reduction in mortality at 20 mg (HR 0.87, CI 0.82–0.93), no effect at 40 mg (HR 1.03, CI 0.95–1.11) and increased mortality at 80 mg (HR 1.17, CI 1.03–1.33). Naproxen reduced mortality to a similar extent at both doses 250 mg (HR 0.89, CI 0.80–0.98) and 500 mg (HR 0.90, CI 0.84–0.98). Tadalafil reduced mortality at all doses, and most pronounced at 10 mg (HR 0.72, CI 0.58–0.89). Sildenafil, likewise, reduced mortality at all doses, with a more pronounced effect at 50 mg (HR 0.85, CI 0.75–0.96).

**FIGURE 5:**
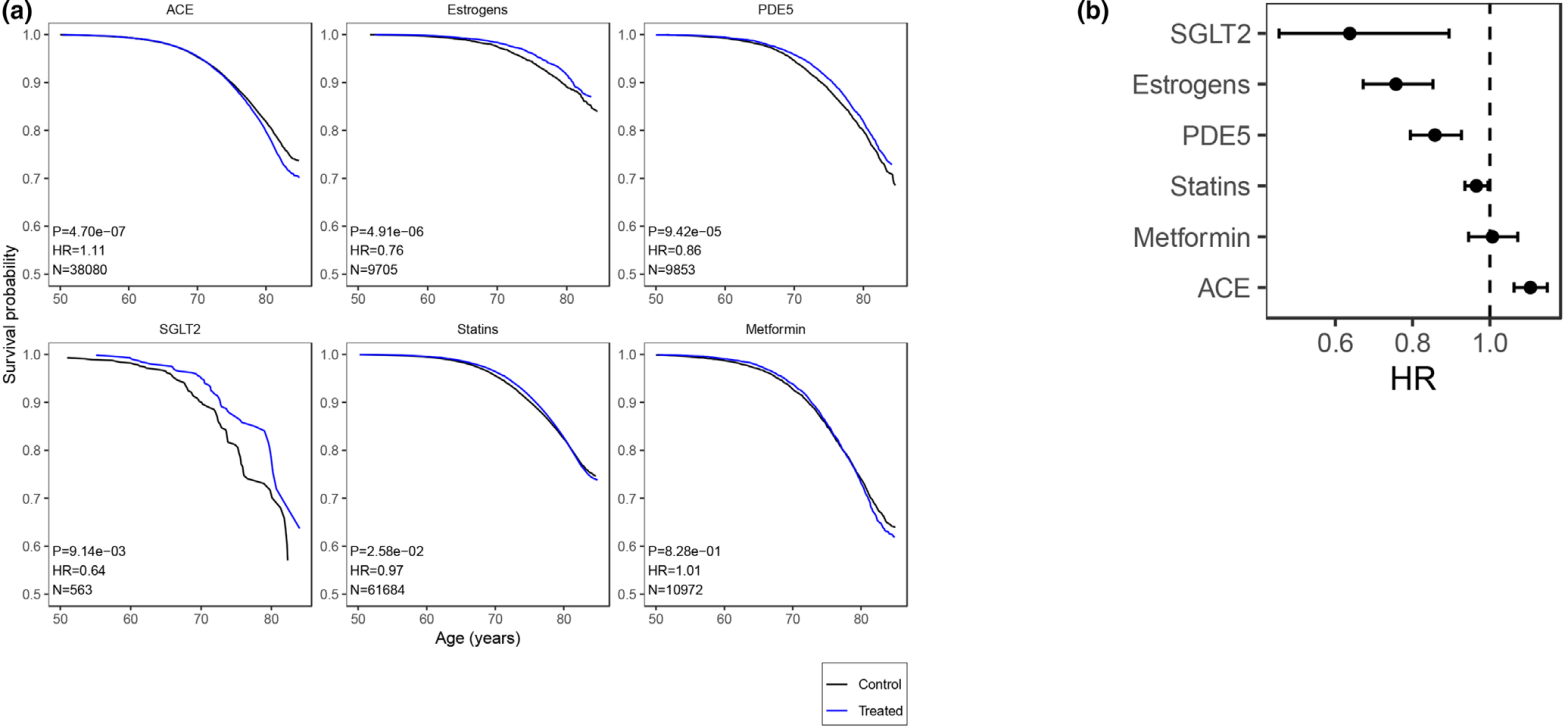
Effect of specific drug classes on mortality. Effect at multiple concentrations (mg) of selected drugs on survival. (a) Survival curves. Survival probability vs. Age (years). N: Sample size (treated), Hazard‐Ratio (HR), >1 for worse survival, *p*‐value of CoxPH model. Treated group in blue, control in black (b) HR with confidence interval 95% in X‐axis. Dose (mg) in Y‐axis. Dotted line HR = 1.

As our findings suggested that there may be a drug class effect on mortality, we performed a pooled analysis, combining drugs from the same class (Methods). We selected Statins, PDE5i, Estrogens, given that we observed some positive effect on survival for some of these drugs. We added SGLT2i, and Metformin, due to previous reports of positive effect, and ACEi due to potential reports of positive effects (Figure 5; Data S5). Statins (HR 0.97, CI 0.94–1.00) and Estrogen (HR 0.76, CI 0.67–0.85), as a class, reduced mortality, so did SGLT2i (HR 0.64, CI 0.45–0.89), although with a much lower sample size. Metformin (HR 1.01, CI 0.95–1.07) had a neutral effect on mortality, while ACEi (HR 1.11, CI 1.06–1.15) was associated with increased mortality.

## 3 DISCUSSION

The present study takes advantage of the large UKBB registry to systematically assess the association between prescribed medications and mortality in the general population. As expected, most prescription drugs are associated with increased mortality. This may in part be driven by the underlying disease the drug is prescribed for, or side effects of the drug. Drugs with increased mortality included opioids (Morphine sulfate, Oramorph), the diuretic Furosemide, pain medication Paracetamol or Tiotropium. The detrimental effect of opioids on lifespan has been documented elsewhere (Wilson et al., 2020), including in the UKBB (Macfarlane et al., 2020). For others, such as widely prescribed Paracetamol, the potential side effect or safety in an older population is still being investigated (Girard et al., 2019). Furosemide is often prescribed in the context of severe conditions, notably heart failure, which may confound its effect. Similarly, Tiotropium, most often prescribed for COPD, may underlie groups with poorer health status. It is further interesting to see two laxative drugs, Senna, and Lactulose, being most associated with increased mortality. Constipation status and laxative use have been independently associated with higher risk of all‐cause mortality and incident CVD (Sumida et al., 2019). Lastly, the association of thiamine with increased mortality may be due to the association between thiamine deficiency and chronic alcoholism (Langlais, 1995).

Conversely, we identified 14 drugs associated with decreased mortality in the general population compared to matched controls. These included notably Atorvastatin, Naproxen, Sildenafil, and Estradiol. Atorvastatin may work by reducing cardiovascular risk. Indeed, it has been proposed that treating cardiovascular risk factors as early as possible in the general population, with either lipid‐lowering medications or anti‐hypertensive drugs, may lead to longer lifespans (Jeemon et al., 2017). Yet the benefits of statins on all‐cause mortality remains largely debated (Collaboration, (CTT), 2010). PDE5i have also been touted as potential pro‐longevity drugs. Notably, in a retrospective study using insurance records, sildenafil was identified to prevent Alzheimer's disease (Adesuyan et al., 2024; Fang et al., 2021). In another study, it was found to prevent CVD and reduce all‐cause mortality (Kloner et al., 2023). Lastly, Estrogen replacement therapy (ERT) have also been reported to have positive effect on mortality in postmenopausal females (Henderson et al., 1991; Sarrel et al., 2013), but there remain active concerns over a potential increased risk of breast cancer (Beral et al., 2019; Cancer, 2019).

Several drugs had been reported in the past to potentially extend lifespan, compared to non‐diseased subjects. In a study from the UK Clinical Practice Research Datalink, metformin‐treated diabetic patients had survival rates comparable to (and, among those age >70, even better than) their matched non‐diabetic control group, even though the diabetic patients were more obese and had greater comorbidities at baseline (Mohammed et al., 2021). Another more recent study found that metformin did not provide survival benefit compared to non‐diseased controls, but SGLT2 inhibitors did (Araldi et al., 2023). In our study Metformin had a neutral effect on lifespan, and SGLT2 inhibitors had a positive impact, but with a lower sample size. Of note, the result on metformin has already been questioned in the past (Mohammed et al., 2021; Stevenson‐Hoare et al., 2023).

Our study has several limitations. A major confounding factor is that the matched controls do not necessarily have the disease the drug was prescribed for in the treated group. Since most drugs are given in the context of a disease that can limit lifespan, their potential for reducing mortality risk in the general population may be underestimated. Indeed, while it is possible that treating disease‐free subjects with the same drugs may increase their lifespan, it was not possible to assess this in the current study. Prospective randomized clinical trials will be necessary to answer this question. Moreover, the results of our study are purely based on retrospective data, and may therefore be confounded by external factors, not recorded in the study. Although we tried to implement an optimal matching strategy, we cannot exclude that for some drugs the matching was not perfect, and therefore control and treated groups did not have the exact same characteristics.

Additionally, despite the large size of the UKBB, for some drugs, we were underpowered to detect the effect on mortality as the number of patients receiving the drug was too low. Furthermore, for more recent drug prescriptions, the drug may not have had enough years on the market to be able to influence mortality risk. Moreover, the criteria we used to consider patients taking the drug (more than 3 months between first and last use) allows for drugs being taken intermittently, or for discontinuation of the drug after a 3‐month period. Lastly, medication recording in the UKBB was stopped after 2017 and the last few years were missing from our study.

Our study also has several strengths including the large sample size of the UKBB, and the ability the measure the effect on mortality of many drugs at the same time in the same cohort. Another strength was the detailed documentation of key confounding factors, including smoking status, cancer diagnosis, diabetes, gender, age at recruitment, which were used for adjustment in our analyses. Moreover, results of dose‐dependent effects, gender‐specific effects, and class effect of drugs strengthen the findings of the study.

As the effect of medications on mortality might differ between countries, it would be interesting to validate these results by performing similar analyses in health registries from other countries. As certain biological mechanisms can improve health early in life but impair it later (Williams, 1957; Zhang et al., 2021), it may be interesting to also investigate age dynamics of the observed associations. Drug interactions could also be explored as part of future studies but may be hampered by low sample size. Similarly, associations between drug prescription and particular cause of death could be explored. Lastly, the benefits on lifespan observed for several drugs in this retrospective study could only be truly determined by performing randomized controlled trials (RCT). Such studies would aim at treating a healthy aging population with a drug during a sufficiently long follow‐up to be able to observe the effect on all‐cause mortality. The Targeting Aging with Metformin (TAME) trial (Barzilai et al., 2016) has been proposed for many years based on such concept, but to our knowledge this trial has not started yet, nor has any other trial of this kind.

## 4 METHODS

### 4.1 Dataset

The data from UKBB was retrieved on October 24, 2023. The fields selected from the participant table were Participant ID, Date of attending assessment center, Age at recruitment, Year of birth, Month of birth, Age at Death, Date of Death, Underlying primary cause of death, Sex, Genetic principal components 1–10, Summed MET minutes per week for all activity, Body mass index BMI, Townsend deprivation index at recruitment, Pack years of smoking, Smoking status, Sleep duration, Cancer diagnosed by doctor, Fractured broken bones in last 5 years, Other serious medical condition/disability diagnosed by doctor, Vascular/heart problem diagnosed by doctor, Blood clot/DVT/bronchitis/emphysema/asthma/rhinitis/eczema/allergy diagnosed by doctor, Diabetes diagnosed by doctor, Age diabetes diagnosed, Alcohol intake frequency, Diagnoses—ICD10. The prescription data was obtained from the Primary Care Linked Data and the fields selected from the prescription data were Participant ID, Date prescription was issued and Drug name.

### 4.2 Inclusion/exclusion of covariates

For these fields to be used in the covariate analysis, they were to present one of two shapes: binary and quantitative covariate. All the fields representing comorbidities at recruitment were transformed into binary covariates (is affected or not affected by this comorbidity). All the fields with quantitative values were left as is. Finally, more complex fields like smoking status were transformed into multiple binary covariates (current smoker and never smoked) and alcohol intake frequency was transformed into monthly alcohol intake, following the mean value of the corresponding code.

Out of all of these, the fields with the most missing data were Pack years of smoking and Summed MET minutes per week for all activity. A univariate survival analysis showed that these covariates had a very low impact on the survival rate, and they were excluded.

### 4.3 Inclusion/exclusion of participants

We censored every participant entry where one of the covariates was missing, as described in the covariate analysis section. Every participant whose death was self‐inflicted or the result of an accident (ICD10 codes starting with O, Q, S, T, V, W, X, Y) were also censored. From the 501′169 starting participants of the UKBB, we were left with 480′444.

### 4.4 Covariate analysis

The covariates selected for the analysis were Current smoker, Cancer diagnosed, Age at recruitment, Diabetes diagnosed, Sex (is male), Other serious medical condition / disability, Vascular / heart problems diagnosed, Never smoked, Townsend deprivation index, Fractured broken bones in last 5 years, Blood clot/DVT/bronchitis/emphysema/asthma/rhinitis/eczema/allergy diagnosed, Body mass index BMI, Alcohol intake monthly frequency, Genetic principal components 1–10, Sleep duration.

We conducted a Cox Proportional Hazards (CoxPH) multivariate survival analysis and classified the covariates under three categories depending on their effect on the survival rate. With a hazard ratio greater than 1.5, covariates “Current smoker”, “Cancer diagnosed”, “Age at recruitment”, “Diabetes diagnosed”, and Sex (is male) were called high‐priority covariates. With a hazard ratio greater than 1.1, covariates “Current smoker”, “Cancer diagnosed”, “Age at recruitment”, “Diabetes diagnosed”, and Sex (is male) were called high‐priority covariates Other serious medical condition/disability, “Vascular/heart problems diagnosed”, “Never smoked”, “Townsend deprivation index”, “Fractured broken bones in last 5 years”, “Blood clot/DVT/bronchitis/emphysema/asthma/rhinitis/eczema allergy diagnosed” were called intermediate‐priority covariates. All the others were called low‐priority covariates.

### 4.5 Matching

Subjects that were prescribed the drug of interest for at least three consecutive months were individually matched with subjects that were not prescribed that drug (or prescribed for less than 3 months) using an algorithm called Nearest Neighbor Covariate Matching (NNCM) (Austin, 2014; Stuart, 2010; Szekér & Vathy‐Fogarassy, 2020). Given how important the different covariates are on the survival rate of individuals, this matching attempts to make sure that the individuals with prescription are compared to individuals presenting a similar subset of covariates.

The covariates were assigned different levels of priority (defined in the covariate analysis), relative to their impact on the survival rate of individuals. The distance metric used in the matching algorithm was adapted to give more weight to the covariates with a higher priority. In other words, the higher the priority on a covariate, the stricter the matching will be relative to that covariate.

Example of this matching is presented in the appendix (Figure S2). The difference in matching between higher priority covariates and lower priority ones can clearly been seen. To achieve an optimal matching strategy, we decided to include all the participants in the cohort, includes ones for which no prescription data was available. We cannot exclude that some of the matched participants were also being prescribed the drug in question.

### 4.6 Drug name curation

A challenge encountered with the drug prescription table is that the same drug can be presented under several different names. An example of this with Omeprazole was the presence of at least 20 variants of “Omeprazole 20mg capsules,” such as “Omeprazole Cap 20mg” or “Omeprazole Multiple Unit Pellet System Dispersible Tablets 20mg,”

To circumvent this issue, two sets of some techniques were used. First automatic techniques such as capitalization and the removal of special characters. Then finally, the remaining drug names were curated matched “by hand,” This method allowed to not only study the effect of drugs in general but also to study the effect of the drugs by concentration.

We limited our analysis to the top 1000 most used drugs in the raw prescribed data after the first automatic round of curation. The second round of handmade name curation brought that number down to 543 unique drugs and 829 unique drugs‐concentration couples. Finally, it was decided to only keep the drugs that were prescribed to at least 500 patients for at least 3 months, which gave the final number of 406 drugs.

### 4.7 Survival analysis

The survival analysis was obtained by comparing two groups for each drug. The first group is comprised of individuals that have been using the drug of interest for at least 3 months. The second group is matched individuals that haven't been using the drug of interest. The results of the survival analysis were obtained by conducting a univariate CoxPH analysis on these two groups.

We conducted three different analyses. The first was on the drug use regardless of concentration, the second one was on the drug and their specific concentration, and the last one was on specific drug groups. For each of these, we analyzed the results on the whole prescription group as well as on the male patients only and female patients only.

The specific drug groups for the third analysis are statins, with Atorvastatin, Fluvastatin, Pravastatin, Rosuvastatin and Simvastatin; SGLT2 inhibitors (Canagliflozin, Empagliflozin, Dapagliflozin); PDE5 inhibitors, with Tadalafil, Sildenafil and Vardenafil; ACE inhibitors, with Captopril, Enalapril, Fosinopril, Lisinopril, Perindopril, Ramipril and Trandolapril; Metformin; Estrogens, with Estraderm, Estradiol, Estriol and Vagifem.

### 4.8 Multiple comparison correction

Assessing significance of results on a multiple comparisons problem can be challenging since having a low threshold for the *p*‐value might not be enough to dodge type I errors (Benjamini, 2010) For these survival analyses, we decided to implement the Benjamini–Hochberg procedure (Benjamini & Hochberg, 1995) with a control level alpha equal to 0.05.

## AUTHOR CONTRIBUTIONS

J. M. performed data and statistical analysis, visualization and co‐wrote the manuscript. Y. R. and H. B. F critically revised the manuscript. A. O. co‐supervised the work, designed the study, and co‐wrote the manuscript. K. P. co‐supervised the work, designed the study, performed data analysis, and co‐wrote the manuscript.

## FUNDING INFORMATION

UK Biobank was established by the Wellcome Trust, Medical Research Council, Department of Health, Scottish government, and Northwest Regional Development Agency. It has also had funding from the Welsh assembly government and the British Heart Foundation. The data access and analyses in this study were funded by EPITERNA.

## CONFLICT OF INTEREST STATEMENT

UK Biobank was established by the Wellcome Trust medical charity, Medical Research Council, Department of Health, Scottish government, and Northwest Regional Development Agency. EPITERNA is a private for‐profit entity, but with no outstanding financial interest in the presented prescription drugs.

## Supporting information


Data S1.

Data S2.

## Data Availability

Data access was granted as “Approved Research” study from the UK biobank, principal investigator: Dr Kevin Perez (EPITERNA SA), Approved Research ID: 107065, Approval date: August 17th, 2023. All data produced in the present work are contained in the manuscript. No additional data available.

## References

[acel14334-bib-0001] Adesuyan, M. , Jani, Y. H. , Alsugeir, D. , Howard, R. , Ju, C. , Wei, L. , & Brauer, R. (2024). Phosphodiesterase type 5 inhibitors in men with erectile dysfunction and the risk of Alzheimer disease: A cohort study. Neurology, 102, e209131. 38324745 10.1212/WNL.0000000000209131 PMC10890837

[acel14334-bib-0002] Andersson, T. , Nåtman, J. , Mourtzinis, G. , Bager, J. E. , Bengtsson Boström, K. , Franzén, S. , & Hjerpe, P. (2023). The effect of statins on mortality and cardiovascular disease in primary care hypertensive patients without other cardiovascular disease or diabetes. European Journal of Preventive Cardiology, 30, 1883–1894. 37368941 10.1093/eurjpc/zwad212

[acel14334-bib-0003] Araldi, E. , Jutzeler, C. R. , & Ristow, M. (2023). Effects of antidiabetic drugs on mortality risks in individuals with type 2 diabetes: A prospective cohort study of UK biobank participants. medRxiv, 2023.05.19.23290214. 10.1101/2023.05.19.23290214

[acel14334-bib-0004] Austin, P. C. (2014). A comparison of 12 algorithms for matching on the propensity score. Statistics in Medicine, 33, 1057–1069. 24123228 10.1002/sim.6004 PMC4285163

[acel14334-bib-0005] Bannister, C. A. , Holden, S. E. , Jenkins‐Jones, S. , Morgan, C. L. , Halcox, J. P. , Schernthaner, G. , Mukherjee, J. , & Currie, C. J. (2014). Can people with type 2 diabetes live longer than those without? A comparison of mortality in people initiated with metformin or sulphonylurea monotherapy and matched, non‐diabetic controls. Diabetes, Obesity & Metabolism, 16, 1165–1173. 10.1111/dom.12354 25041462

[acel14334-bib-0006] Barzilai, N. , Crandall, J. P. , Kritchevsky, S. B. , & Espeland, M. A. (2016). Metformin as a tool to target aging. Cell Metabolism, 23, 1060–1065. 27304507 10.1016/j.cmet.2016.05.011 PMC5943638

[acel14334-bib-0007] Benjamini, Y. (2010). Simultaneous and selective inference: Current successes and future challenges. Biometrical Journal, 52, 708–721. 21154895 10.1002/bimj.200900299

[acel14334-bib-0008] Benjamini, Y. , & Hochberg, Y. (1995). Controlling the false discovery rate: A practical and powerful approach to multiple testing. Journal of the Royal Statistical Society: Series B (Methodological), 57, 289–300.

[acel14334-bib-0009] Beral, V. , Peto, R. , Pirie, K. , & Reeves, G. (2019). Menopausal hormone therapy and 20‐year breast cancer mortality. Lancet, 394, 1139. 31474331 10.1016/S0140-6736(19)32033-1

[acel14334-bib-0010] Cancer, C. G. on H. F. in B . (2019). Type and timing of menopausal hormone therapy and breast cancer risk: Individual participant meta‐analysis of the worldwide epidemiological evidence. Lancet, 394, 1159–1168. 31474332 10.1016/S0140-6736(19)31709-X PMC6891893

[acel14334-bib-0011] Chang, T. I. , Park, H. , Kim, D. W. , Jeon, E. K. , Rhee, C. M. , Kalantar‐Zadeh, K. , Kang, E. W. , Kang, S. W. , & Han, S. H. (2020). Polypharmacy, hospitalization, and mortality risk: A nationwide cohort study. Scientific Reports, 10, 18964. 33144598 10.1038/s41598-020-75888-8 PMC7609640

[acel14334-bib-0012] Collaboration, C. T. T. (CTT) , et al. (2010). Efficacy and safety of more intensive lowering of LDL cholesterol: A meta‐analysis of data from 170 000 participants in 26 randomised trials. Lancet, 376, 1670–1681. 21067804 10.1016/S0140-6736(10)61350-5 PMC2988224

[acel14334-bib-0013] Cristea, M. , Noja, G. G. , Stefea, P. , & Sala, A. L. (2020). The impact of population aging and public health support on EU labor markets. International Journal of Environmental Research and Public Health, 17, 1439. 32102277 10.3390/ijerph17041439 PMC7068414

[acel14334-bib-0014] Davies, L. E. , Kingston, A. , Todd, A. , & Hanratty, B. (2022). Is polypharmacy associated with mortality in the very old: Findings from the Newcastle 85+ study. British Journal of Clinical Pharmacology, 88, 2988–2995. 34981552 10.1111/bcp.15211 PMC9302636

[acel14334-bib-0015] Fang, J. , Zhang, P. , Zhou, Y. , Chiang, C. W. , Tan, J. , Hou, Y. , Stauffer, S. , Li, L. , Pieper, A. A. , Cummings, J. , & Cheng, F. (2021). Endophenotype‐based in silico network medicine discovery combined with insurance record data mining identifies sildenafil as a candidate drug for Alzheimer's disease. Nature Aging, 1, 1175–1188. 35572351 10.1038/s43587-021-00138-z PMC9097949

[acel14334-bib-0016] Girard, P. , Sourdet, S. , Cantet, C. , Barreto, P. , De, S. , & Rolland, Y. (2019). Acetaminophen safety: Risk of mortality and cardiovascular events in nursing home residents, a prospective study. Journal of the American Geriatrics Society, 67, 1240–1247. 30912588 10.1111/jgs.15861

[acel14334-bib-0017] Hasan, F. , & Hasan, B. (2017). Antidiabetic medications and mortality reduction: A shift from surrogate to clinical endpoints. Therapeutic Advances in Endocrinology and Metabolism, 8, 173–174. 29238516 10.1177/2042018817737955 PMC5721968

[acel14334-bib-0018] Henderson, B. E. , Paganini‐Hill, A. , & Ross, R. K. (1991). Decreased mortality in users of estrogen replacement therapy. Archives of Internal Medicine, 151, 75–78. 1985611

[acel14334-bib-0019] Jeemon, P. , Gupta, R. , Onen, C. , Adler, A. , Gaziano, T. A. , Prabhakaran, D. , & Poulter, N. (2017). Chapter 22: Management of hypertension and dyslipidemia for primary prevention of cardiovascular disease. In D. Prabhakaran , S. Anand , T. A. Gaziano , J. C. Mbanya , Y. Wu , & R. Nugent (Eds.), Cardiovascular, respiratory, and related disorders (3rd ed.). The International Bank for Reconstruction and Development/The World Bank. https://www.ncbi.nlm.nih.gov/books/NBK525163/ 30212083

[acel14334-bib-0020] Kloner, R. A. , Stanek, E. , Crowe, C. L. , Singhal, M. , Pepe, R. S. , Bradsher, J. , & Rosen, R. C. (2023). Effect of phosphodiesterase type 5 inhibitors on major adverse cardiovascular events and overall mortality in a large nationwide cohort of men with erectile dysfunction and cardiovascular risk factors: A retrospective, observational study based on healthcare claims and national death index data | the journal of sexual medicine|Oxford academic. The Journal of Sexual Medicine, 20, 38–48. 36897243 10.1093/jsxmed/qdac005

[acel14334-bib-0021] Kulkarni, A. S. , Aleksic, S. , Berger, D. M. , Sierra, F. , Kuchel, G. A. , & Barzilai, N. (2022). Geroscience‐guided repurposing of FDA‐approved drugs to target aging: A proposed process and prioritization. Aging Cell, 21, e13596. 35343051 10.1111/acel.13596 PMC9009114

[acel14334-bib-0022] Langlais, P. J. (1995). Alcohol‐related thiamine deficiency: Impact on cognitive and memory functioning. Alcohol Health and Research World, 19, 113–121. 31798071 PMC6875731

[acel14334-bib-0023] Li, J.‐R. , Yeh, L.‐L. , Lin, J.‐Y. , & Pan, Y.‐J. (2024). Medication dosage impact on mortality in old‐age individuals with schizophrenia: A National Cohort Study. Pharmaceuticals, 17, 78. 38256911 10.3390/ph17010078 PMC10820447

[acel14334-bib-0024] Macfarlane, G. J. , Beasley, M. , Jones, G. T. , & Stannard, C. (2020). The epidemiology of regular opioid use and its association with mortality: Prospective cohort study of 466 486 UK biobank participants. EClinicalMedicine, 21, 100321. 32322808 10.1016/j.eclinm.2020.100321 PMC7170951

[acel14334-bib-0025] McNeil, J. J. , et al. (2018). Effect of aspirin on all‐cause mortality in the healthy elderly. The New England Journal of Medicine, 379, 1519–1528. 30221595 10.1056/NEJMoa1803955 PMC6433466

[acel14334-bib-0026] Miller, K. D. , et al. (2022). Cancer treatment and survivorship statistics, 2022. Cancer Journal Clinical, 72, 409–436. 10.3322/caac.21731 35736631

[acel14334-bib-0027] Mohammed, I. , Hollenberg, M. D. , Ding, H. , & Triggle, C. R. (2021). A critical review of the evidence that metformin is a putative anti‐aging drug that enhances Healthspan and extends lifespan. Frontiers in Endocrinology, 12, 718942. 34421827 10.3389/fendo.2021.718942 PMC8374068

[acel14334-bib-0028] Nowak, M. M. , Niemczyk, M. , Florczyk, M. , Kurzyna, M. , & Pączek, L. (2022). Effect of statins on all‐cause mortality in adults: A systematic review and meta‐analysis of propensity score‐matched studies. Journal of Clinical Medicine, 11, 5643. 36233511 10.3390/jcm11195643 PMC9572734

[acel14334-bib-0029] Rolland, Y. , Sierra, F. , Ferrucci, L. , Barzilai, N. , de Cabo, R. , Mannick, J. , Oliva, A. , Evans, W. , Angioni, D. , de Souto Barreto, P. , Raffin, J. , Vellas, B. , Kirkland, J. L. , the G.C.T‐TF group , Andrieu, S. , Bacqueville, D. , Bischoff‐Ferrari, H. , Blivet, G. , Cash, T. , … Zhavoronkov, A. (2023). Challenges in developing Geroscience trials. Nature Communications, 14, 5038. 10.1038/s41467-023-39786-7 PMC10439920 37598227

[acel14334-bib-0030] Sarrel, P. M. , Njike, V. Y. , Vinante, V. , & Katz, D. L. (2013). The mortality toll of estrogen avoidance: An analysis of excess deaths among hysterectomized women aged 50 to 59 years. American Journal of Public Health, 103, 1583–1588. 23865654 10.2105/AJPH.2013.301295 PMC3780684

[acel14334-bib-0031] Sierra, F. (2016). The emergence of Geroscience as an interdisciplinary approach to the enhancement of health span and life span. Cold Spring Harbor Perspectives in Medicine, 6, a025163. 26931460 10.1101/cshperspect.a025163 PMC4817738

[acel14334-bib-0032] Stevenson‐Hoare, J. , Leonenko, G. , & Escott‐Price, V. (2023). Comparison of long‐term effects of metformin on longevity between people with type 2 diabetes and matched non‐diabetic controls. BMC Public Health, 23, 804. 37131166 10.1186/s12889-023-15764-y PMC10155360

[acel14334-bib-0033] Stuart, E. A. (2010). Matching methods for causal inference: A review and a look forward. Statistical Science, 25, 1–21. 20871802 10.1214/09-STS313 PMC2943670

[acel14334-bib-0034] Sumida, K. , Molnar, M. Z. , Potukuchi, P. K. , Thomas, F. , Lu, J. L. , Yamagata, K. , Kalantar‐Zadeh, K. , & Kovesdy, C. P. (2019). Constipation and risk of death and cardiovascular events. Atherosclerosis, 281, 114–120. 30658186 10.1016/j.atherosclerosis.2018.12.021 PMC6399019

[acel14334-bib-0035] Szekér, S. , & Vathy‐Fogarassy, Á. (2020). Weighted nearest neighbours‐based control group selection method for observational studies. PLoS One, 15, e0236531. 32701991 10.1371/journal.pone.0236531 PMC7377436

[acel14334-bib-0036] Tiihonen, J. , Mittendorfer‐Rutz, E. , Torniainen, M. , Alexanderson, K. , & Tanskanen, A. (2016). Mortality and cumulative exposure to antipsychotics, antidepressants, and benzodiazepines in patients with schizophrenia: An observational follow‐up study. The American Journal of Psychiatry, 173, 600–606. 26651392 10.1176/appi.ajp.2015.15050618

[acel14334-bib-0037] Williams, G. C. (1957). Pleiotropy, natural selection, and the evolution of senescence. Evolution, 11, 398.

[acel14334-bib-0038] Wilson, N. , Kariisa, M. , Seth, P. , Smith, H. , & Davis, N. L. (2020). Drug and opioid‐involved overdose deaths — United States, 2017–2018. Morbidity and Mortality Weekly Report, 69, 290–297. 32191688 10.15585/mmwr.mm6911a4 PMC7739981

[acel14334-bib-0039] Zhang, W. B. , Ye, K. , Barzilai, N. , & Milman, S. (2021). The antagonistic pleiotropy of insulin‐like growth factor 1. Aging Cell, 20, e13443. 34363732 10.1111/acel.13443 PMC8441393

