# Supplementary material for: Association between prescription drugs and all‐cause mortality risk in the UK population
Source: Aging Cell. 2024 Oct 4;23(12):e14334. doi: 10.1111/acel.14334 (PMC11634711; doi:10.1111/acel.14334)
Supplement: Supplementary file 2 — Data S2. [file ACEL-23-e14334-s002.zip › Supplementry files.docx]

**A**

Figure S1. **Effect of main covariates on mortality.** Effect if selected covariates on survival. (**A**) Survival curves. Survival probability vs. Age (years). N: sample size (treated), Hazard-Ratio (HR), > 1 for worse survival, P-value of CoxPH model. Treated group in blue, control in black (**B**) HR with confidence interval 95% in x-axis for top covariates. Dotted line HR = 1.

**B**

Blood clot, DVT, bronchitis, emphysema

**A**

**B**

Figure S2. **NNCM matching results.** (**A**) Matching of prescription drugs associated with decreased lifespan. (**B**) Matching of prescription drugs associated with increased lifespan. Whole cohort in red, treated group in green, matched control group in blue.

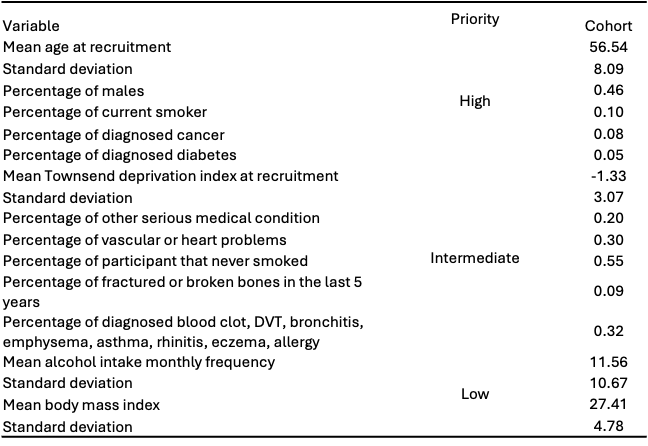

Table S2. **Description of the drugs most and least associated with increased mortality.** Drug name, list of ingredients, association with mortality, route of administration, primary indications.

Table S1. **Cohort characteristics.** Main covariates, whole cohort.
